# Molecular Basis for Antioxidant Enzymes in Mediating Copper Detoxification in the Nematode *Caenorhabditis elegans*

**DOI:** 10.1371/journal.pone.0107685

**Published:** 2014-09-22

**Authors:** Shaojuan Song, Xueyao Zhang, Haihua Wu, Yan Han, Jianzhen Zhang, Enbo Ma, Yaping Guo

**Affiliations:** 1 Institute of Applied Biology, Shanxi University, Taiyuan, Shanxi, China; 2 School of Life Science, Shanxi University, Taiyuan, Shanxi, China; NIEHS/NIH, United States of America

## Abstract

Antioxidant enzymes play a major role in defending against oxidative damage by copper. However, few studies have been performed to determine which antioxidant enzymes respond to and are necessary for copper detoxification. In this study, we examined both the activities and mRNA levels of SOD, CAT, and GPX under excessive copper stress in *Caenorhabditis elegans*, which is a powerful model for toxicity studies. Then, taking advantage of the genetics of this model, we assessed the lethal concentration (LC_50_) values of copper for related mutant strains. The results showed that the SOD, CAT, and GPX activities were significantly greater in treated groups than in controls. The mRNA levels of *sod-3*, *sod-5*, *ctl-1*, *ctl-2*, and almost all *gpx* genes were also significantly greater in treated groups than in controls. Among tested mutants, the *sod-5*, *ctl-1*, *gpx-3*, *gpx-4*, and *gpx-6* variants exhibited hypersensitivity to copper. The strains with SOD or CAT over expression were reduced sensitive to copper. Mutations in *daf-2* and *age-1*, which are involved in the insulin/insulin-like growth factor-1 signaling pathway, result in reduced sensitivity to stress. Here, we showed that LC_50_ values for copper in *daf-2* and *age-1* mutants were significantly greater than in N2 worms. However, the LC_50_ values in *daf-16;daf-2* and *daf-16;age-1* mutants were significantly reduced than in *daf-2* and *age-1* mutants, implying that reduced copper sensitivity is influenced by DAF-16-related functioning. SOD, CAT, and GPX activities and the mRNA levels of the associated copper responsive genes were significantly increased in *daf-2* and *age-1* mutants compared to N2. Additionally, the activities of SOD, CAT, and GPX were greater in these mutants than in N2 when treated with copper. Our results not only support the theory that antioxidant enzymes play an important role in copper detoxification but also identify the response and the genes involved in these processes.

## Introduction

Copper is an essential metal but is toxic at high doses. Elevated copper contamination levels resulting from human activities have been widely documented [1], [2]. Currently, the field of toxicology is moving away from the measurement of single endpoints (such as growth or mortality) and toward measurements of how organisms respond to toxic exposures at the cellular level [3]–[6]. Similarly, copper toxicology is moving from studying conditions of acute toxicity (such as growth, behavior or mortality) and toward investigating how organisms respond to copper toxicity [3], [5], [7], [8]. Copper toxicity results from the accumulation of oxidative damage generated by reactive oxygen species via Fenton-like reaction processes [9]–[12]. Therefore, the induction of antioxidant enzymes is an important protective mechanism that minimizes organisms' oxidative damage from copper [13]. The malondialdehyde (MDA), hydrogen peroxide (H_2_O_2_) and superoxide (O_2_^’−^) contents are sharply increased, which subsequently enhances the antioxidant enzyme activities of superoxide dismutase (SOD), glutathione peroxidase (GPX), catalase (CAT), and glutathione *S*-transferase (GST) [14], [15]. However, the molecular mechanisms underlying copper detoxification are still poorly understood in nematodes, despite the fact that copper is a dominant contaminant in the environment.

*Caenorhabditis elegans*, an excellent experimental model organism, is well -suited to the investigation of toxicological processes (due to its short life cycle, low cost, sequenced genome, and ease with which mass cultures can be generated) [16]–[19]. Other advantages, including the conservation of stress response pathways, availability of mutant and transgenic strains, and wealth of biological information, have led to the increased use of *C.elegans* in toxicological studies [20]–[22]. The effects of copper toxicity (such as mortality, growth, behavior, and brood size) have been commonly reported in *C. elegans* [23]–[25]. These features make the nematode a user-friendly animal for the study of toxicity. Although we previously reported that elevated copper levels produce oxidative damage in the worms [26], [27], detailed information concerning the activation of the antioxidant system in the detoxification of copper is limited, and few analyses have been conducted to determine the antioxidant genes are necessary for such detoxification.

Particularly, Genes associated with the insulin/IGF signaling pathway constitutively enhance the activities of stress-defense-related enzymes, such as SOD and CAT, causing hyposensitivity to heat, UV, oxidants and heavy metals [28]–[30]. In this insulin-like signaling pathway, *age-1* encodes phosphatidylinositol-3-kinase and transmits signals from *daf-2*, the insulin receptor-like protein, into the cell [31]. This hyposensitivity may be mediated by *daf-16*, a forkhead transcription factor [29]. At the same time, the expression of *sod-1*, *sod-3*, *sod-5*, *ctl-1*, and *ctl-2* is regulated directly or indirectly by this signaling pathway [29], [32]. Hyposensitivity to copper does not appear to be correlated with the expression of metallothioneins [28]. However, little evidence has directly indicated that antioxidant enzymes are necessary in the copper detoxification process.

The aims of this work are the following: (I) to identify which antioxidant enzymes and associated genes play a key role in copper detoxification and (II) to determine the antioxidant enzymes that mediate copper response capacities in *daf-2*, *age-1* and *daf-16* mutants. Our study will contribute to the understanding of the molecular detoxification mechanisms of antioxidant enzymes induced by copper.

## Materials and Methods

### *C. elegans* strains: culture and maintenance

Worm culture, maintenance and synchronization were performed as previously described [33]. The strains used in this study were N2, *sod-1(tm776)*, *sod-2(gk257)*, *sod-3(tm760)*, *sod-4(gk101)*, *sod-5(tm1146)*, *ctl-1(ok1242)*, *ctl-2(ok1137)*, *ctl-3(ok2042)*, *wuIs151*(*ctl-1*+*ctl-2*+*ctl*-*3*+*myo*-2::GFP), *wuIs152(sod-1 gDNA)*, *wuIs156(sod-2 gDNA)*, *huIs33(sod-3 gDNA)*, *daf-16(m26)*, *age-1(hx546)*, *daf-2(e1368)*, *daf-16(m26);age-1(m333)*, and *daf-16(m26);daf-2(e1370)*, which were provided by the Caenorhabditis Genetics Center (CGC), as well as *gpx-1(tm2100), gpx-2(tm2895), gpx-3(tm2139), gpx-4(tm2111), gpx-5(tm2042), gpx-6(tm2535), gpx-7(tm2166),* and *gpx-8(tm2108),* which were provided by the National Bioresource Project (NBRP).

### Chemicals and reagents

CuSO_4_·5H_2_O was used as an analytical reagent and was obtained from Sigma-Aldrich, St. Louis, MO, USA and Shanghai Zhizhen Chemical Co., Ltd., purity ≥99%. All other chemicals and reagents used in this study were of molecular biology grade and were purchased from Sigma Chemicals (St. Louis, Mo, USA), Qiagen (Valencia, CA, USA) or Invitrogen (Carlsbad, CA, USA).

### Copper exposure for enzyme assays

CuSO_4_·5H_2_O was dissolved in ultra-pure distilled water. A uracil-deficient strain of *E.coli*, OP50, was cultured in L-Broth (3 g yeast extract, 10 g tryptone and 10 g NaCl in 1 L of ddH_2_O) as a food source. One liter of saturated OP50 was centrifuged and resuspended in 100 mL K -medium (32 mmol/L KCl and 52 mmol/L NaCl). This wash procedure was repeated three times. The 3-day-old worms (young adults) were transferred to fresh K -medium [34], which was supplemented with different concentrations of CuSO_4_ (0, 0.05, 0.1, 0.2, 0.4, or 0.8 mM). A volume of OP50 equal to 1/10 of the test solution was added. No experimental conditions, including CuSO_4_ concentrations, induced mortality in *C. elegans*. The assays were performed in disposable borosilicate tubes with constant shaking at 20°C. The tubes were tilted on a supported surface with constant shaking for 24±1 h at 20°C.

To confirm the results, we also conducted exposure experiments as follows: the L4 populations were transferred into K medium containing 0.8 mM CuSO_4_ with bacteria. The assays were performed in 300 mL flasks for 24 h with 50 µmol FUDR (5-Fluoro-2-deoxyuridine, Sigma) at 150 rpm and 20°C. The worms were then collected by centrifugation and washed. Live worms were separated by sucrose flotation [35].

### Antioxidant enzyme activities

After exposure to 0, 0.05, 0.1, 0.2, 0.4, or 0.8 mmol/L CuSO_4_ for 24 h, worms were repeatedly collected and cleaned at 825×g for 1 min. The worms were suspended in cold homogenized buffer (0.01 mol/L Tris-HCl, 0.0001 mol/L EDTA-2Na, 0.01 mol/L sucrose, 0.8% NaCl, according to the protocol of the Nanjing Jiancheng Bioengineering Institute) and homogenized using a homogenizer for 6 min on ice. The mixture was centrifuged at 367×g for 10 min at 4°C. The upper aqueous layer containing the enzyme was transferred to a new 1.5 mL EP tube for an enzymatic assay. Protein contents were determined using standard methods (bicinchoninic acid), as described previously [36].

Superoxide dismutase (SOD) activity in worms was measured using a commercial chemical assay kit (Nanjing Jiancheng Bioengineering Institute, Nanjing, China) by the xanthine oxidase method [37]. SOD is a specific inhibitor of the superoxide anion radical. There are only two SODs, – Cu/Zn-SOD and Mn-SOD–, which make up the total SOD (T-SOD) in nematodes. Mn-SOD activity is lost when treated with regents; however, Cu/Zn-SOD activity is invariant.

Catalase (CAT) activity was measured using ammonium molybdate methods [38] with the assay kit of Nanjing Jiancheng, China. The enzyme extract was incubated with H_2_O_2_ for 1 min at 37°C. The reaction was quickly stopped by the addition of ammonium molybdate. A CAT unit is defined as the decomposition of 1 µmol H_2_O_2_ per second.

Glutathione peroxidase (GPx) activity in worms was evaluated according to the DTNB (5,5'-Dithiobis-(2-nitrobenzoic acid)) method [39] using a colorimetric GSH-Px detection kit (Nanjing Jiancheng, China). Briefly, the homogenate was incubated with H_2_O_2_ and GSH for 5 min at 37°C before 2-thio two nitrobenzoic acid was added to stop the reaction.

GST activity was quantified by the CDNB (2,4-Dinitrochlorobenzene, Shanghai Sangon Biological Engineering Technology, China) method. Fifty microliters of supernatant was used in a total reaction volume of 250 µL. The substrates for GST, CDNB (1 mM) and GSH (5 mM), were added to the reaction wells. The change in absorbance of CDNB conjugate for the first minute was measured at 340 nm and 28°C at 12 -s intervals (*ε*340 = 9.6 mM^−1^ cm^−1^). Nonenzymatic reaction activities were subtracted from the enzymatic activity.

The SOD, CAT, GPX, and GST activities were then measured at an absorbances of 550, 405, 412, or 340 nm, respectively, using a microplate reader and the SOFTmax software (Molecular Devices, Sunnyvale, CA).

### Isolation of RNA and cDNA synthesis

Total RNAs were isolated from *C. elegans* by freeze -cracking (in which RNA isolation suspension is completely frozen in liquid nitrogen and then transfered to a 37°C heating block (ThermoQ, China) to thaw completely; this process is repeated 5 times) [40] and extracted using the conventional RNAiso Plus reagent (TaKaRa, China), according to the manufacturer's instructions. RNA was quantified with a NanoDrop 2000 spectrophotometer (Thermo, USA). Reverse transcription was carried out using the Go Taq 2-Step RT-qPCR System (TaKaRa, China). The cDNA product was stored at −80°C until use.

### Real-time PCR

All of the PCR primers (Table S1) were designed using Primer Premier 5.0. Quantitative real-time PCR (qPCR) was performed using the SYBR Premix Ex Taq (TaKaRa, China), according to the manufacturer's instructions, on a Biosystems 7300 real-time PCR system (Applied Biosystems Inc., Foster, CA, USA) using cDNA at a 1∶20 dilution. A dissociation curve was established for each sample. The cycling conditions were as follows: 3 min at 95°C, 40 cycles of 95°C for 5 s and 60°C for 31 s. Fluorescence data were obtained at the 60°C step. For each primer set, target quantities were derived from standard curves, which were generated using the Ct value for each dilution plotted against the log of its concentration. Relative expression values were calculated by dividing the quantities of the target sequence of interest with the quantity obtained for *cdc-42* (RHO GTPase) and *Y45F10D.4* (an iron -binding protein involved in Fe-S cluster formation) as internal reference genes [41].

### Lethality tests

Experimental design and test procedures were conducted as previously described [42] with minor modifications. All nematode strains were exposed to their assigned concentrations at the L_4_ stage (control and seven increasing CuSO_4_ concentrations) in 24-well tissue culture plates (Corning Incorporated, USA) with food source equal to 1/10 of the test solution for 24 h (±1). Each exposure well contained approximately 30 worms in 0.8 mL test solution, and each treatment consisted of 3 exposure wells for a total of 90 worms per CuSO_4_ concentration. After the 24 h exposure period, the worms were scored under a dissection microscope as alive if moving or dead if unresponsive to gentle probing. Aqueous lethality tests were replicated three times for LC_50_ analysis.

### Statistical analysis

Data were expressed as means±SE. Graphs were generated using SigmaPlot 10.0 software (Systat Software, Inc., USA). The CuSO_4_ LC_50_ was determined with SPSS 11.5 (SPSS Manager Inc., USA). The data were log transformed and subjected to a χ^2^ test for normality and to Bartlett's test for homogeneity. Probit analysis was used to calculate the LC_50_ and confidence interval (CI). Significant variations were determined using Student's *t*-tests, except for Duncan's multiple comparison test as required. Probability levels of 0.05 or less were considered statistically significant. Four replicates for each treatment and control were conducted for enzyme and mRNA assays and three replicates were used for the lethality test.

### Ethics Statement

The work described here has not been published previously (except in the form of an abstract or as part of a published lecture or academic thesis) and, is not under consideration for publication elsewhere. Its publication is approved by all authors and by the responsible authorities (the full name of the group leader is Enbo, Ma) where the work was carried out, and, if accepted, it will not be published elsewhere in the same form, in English or in any other language, including electronically, without the written consent of the copyright-holder.

## Results and Discussion

To investigate the role of antioxidant enzymes in the copper detoxification, we first investigated the effects of copper on the antioxidant enzymes' activities and gene expression levels. The status of copper pollution in China is between 0.4 mM to 26 mM [1], [43]. The young adult nematode, which is more resistant to copper than any other development stage, dies at exposure to concentrations higher than 0.8 mM. If only live at high concentrations in nematodes were examined, resistant selection would confound the results. Accordingly, the exposure concentrations of CuSO_4_ were determined according to the minimum lethality doses of 0, 6.25, 12.5, 25, and 100%. Four enzymes (SOD, CAT, GPX, and GST) were measured after cultivation of the 3-day-old worms (young adults) in the nominal value presence of 0, 0.05, 0.1, 0.2, 0.4, and 0.8 mM CuSO_4_ for 24 h. No mortality was observed in either the control or CuSO_4_-exposed groups. A detailed description of the genes and mutants studied in these studies can be found in table 1.

**Table 1:** Mutant strains screened by the toxicity test.

Genes tested	Genes products
*sod-1(tm776)*	Major Cu/Zn SOD
*sod-2(gk257)*	Major Mn SOD
*sod-3(tm760)*	Minor Mn SOD
*sod-4(gk101)*	Extracellular Cu/Zn SOD
*sod-5(tm1146)*	Minor Cu/Zn SOD
*ctl-1(ok1242)*	Cytocolic catalysis
*ctl-2(ok1137)*	Peroxisomal catalysis
*ctl-3(ok2042)*	Pharyngeal muscles and neuron catalysis
*gpx-1(tm2100)*	phospholipid hydroperoxide Glutathione peroxidase
*gpx-2(tm2895)*	Glutathione peroxidase
*gpx-3(tm2139)*	Glutathione peroxidase
*gpx-4(tm2111)*	Glutathione peroxidase
*gpx-5(tm2024)*	Glutathione peroxidase
*gpx-6(tm2535)*	Glutathione peroxidase
*gpx-7(tm2166)*	Glutathione peroxidase
*gpx-8(tm2108)*	Glutathione peroxidase
*age-1(hx546)*	Catalytic subunit of phosphatidylinositol-3-kinase
*daf-2(e1368)*	Insulin-like growth factor receptor
*daf-16(m26)*	Forkhead transcription factor
*daf-16(m26);age-1(m333)*	AGE-1 lying downstream of the DAF-2/insulin receptor and upstream of DAF-16
*daf-16(m26);daf-2(e1370)*	DAF-2 signals through a conserved PI 3-kinase pathway to negatively regulate the activity of DAF-16

### SOD activity and mRNA expression levels

**Figure 1:**
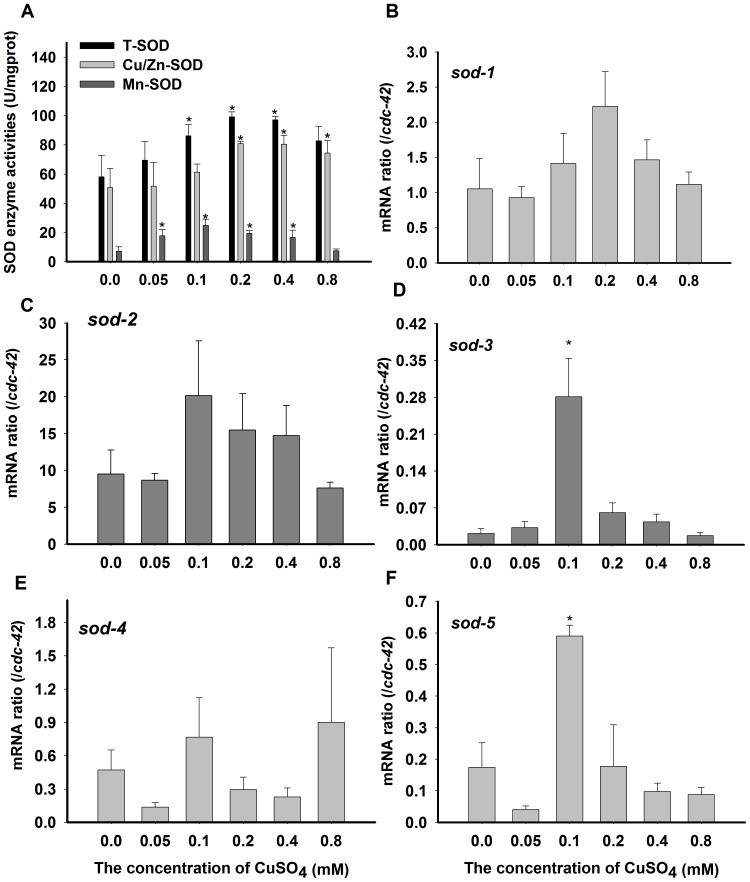
SODs activities or mRNA expression induced by copper in *C. elegans*. (A) SOD activity assessed in nematode N2 at different concentrations of copper exposure. All values are given as the means ±SE (*n* = 3) in U mg^−1^ Pr. The gene expressions of *sod-1*(B), *sod-2* (C), *sod-3* (D), *sod-4* (E), and *sod-5* (F) were quantified in N2 at different concentrations of copper exposure and normalized with *cdc-42*. * *P*<0.05, ** *P*<0.01 versus respective controls analyzed by *t*-test.

Total SOD activity (T-SOD) was significantly increased at all concentrations (from 0.1 to 0.8 mM CuSO_4_) of treatment compared to the untreated group (fig 1A); the results were approximately 1.7-fold greater than those of the controls. We also examined the induction of Cu/Zn-SOD and Mn-SOD. Interestingly, the induction of Cu/Zn-SOD activities was apparent at treatment concentrations of 0.2, 0.4, and 0.8 mmol/L CuSO_4_, which induced activity increases of approximately 1.5-fold, compared to the control group (fig 1A). The induction of Mn-SOD activities was significant based on treatment concentrations of 0.05, 0.1, 0.2, and 0.4 mmol/L CuSO_4_, at which Mn-SOD activities were increased by 2.4-, 3.4-, 2.7, and 2.3-fold, respectively (fig 1A). These data indicate that even as low as 0.05 mmol/L CuSO_4_ may initiate an individual stress response. These increases in SOD activities were also reported with copper treatment [9]. Copper, as a SOD co-factor, may have the ability to increase SOD activities [10].

In *C. elegans*, there are two cytosolic Cu/Zn-SODs encoded by *sod-1* and *sod-5*, one extracellular Cu/Zn-SOD encoded by *sod-4*, and two mitochondrial Mn-SODs encoded by *sod-2* and *sod-3*. The Mn-SOD likely contributes only a minor fraction of total SOD activity (fig 1A). *sod-2* and *sod-1* are highly expressed, whereas *sod-3* and *sod-5* are minor isoforms, the expressions of which are increased in response to stressful situations [44]. In the present study, we quantified the expression levels of each of the SOD genes by qPCR. The results showed that mRNA levels of *sod-3* and *sod-5* were significantly induced (13 and 3-fold greater than the control, respectively) by 0.1 mmol/L CuSO_4_ in the expression of five SOD genes (fig 1C and E). However, neither *sod-3* nor *sod-5* mRNA levels were significantly up-regulated under any other CuSO_4_ treatment concentrations in this study. Other genes were slightly elevated (with no significant difference), which might have contributed to the elevated enzyme activity (fig 1). The increased specificity of the -mRNA levels of *sod* has been observed in other aquatic organisms [14], [45].

### CAT activity and mRNA expression levels

**Figure 2:**
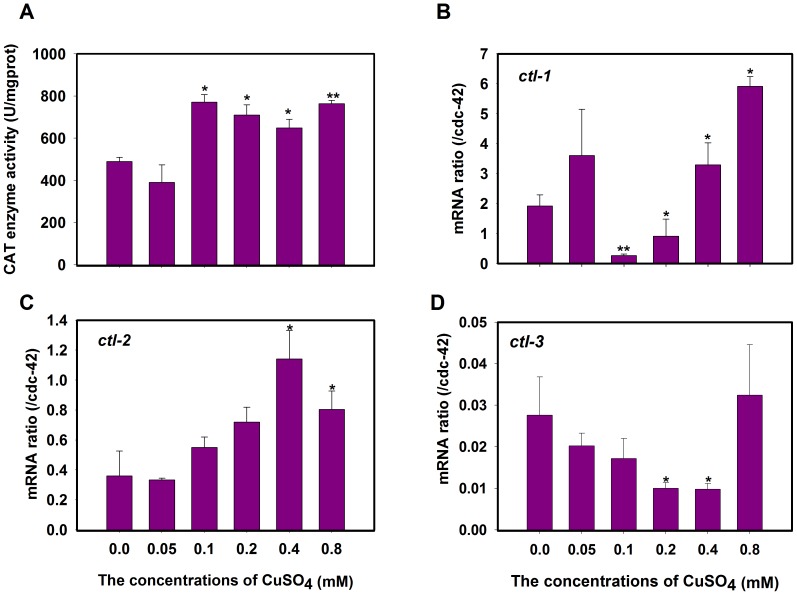
CATs activities or mRNA expression induced by copper in *C. elegans*. (A) The CAT activity and H_2_O_2_ content were changed during the copper stress. Values are presented as the means ±SE (*n* = 3) in U g^−1^ Pr. and mmol mg^−1^ Pr. The gene expressions of *ctl-1*(B), *ctl-2* (C), and *ctl-3* (C) were quantified in nematode N2 under the stress of different concentration of copper and normalized with *cdc-42*. *P<0.05, **P<0.01 versus respective controls analyzed by *t*-test.

Although SODs play a role in preventing oxidative damage [46], the detoxification process is completed by CAT because H_2_O_2_ is a by-product of oxygen metabolism and a substrate of CAT [46]. With respect to CAT activity, treatment with 0.1, 0.2, 0.4, and 0.8 mM concentrations of CuSO_4_ induced a significant increase of 1.3- to 1.6- fold (fig 2A). Such increases in the activities of CAT have been reported with copper treatments [9], [14], [45], and the extent of the increase varied among the treated concentrations and different species [47]. The response of CAT to copper can be thought of as a defensive mechanism that acts with SODs to limit the toxic impact of copper at physiological levels.

In *C.elegans*, three CAT genes have been characterized: *ctl-1*, *ctl-2*, and *ctl-3* [48]. *ctl-1* encodes an unusual cytosolic catalase, the mRNA level of which was increased by approximately 1.7-fold and 3.1-fold in the 0.4 and 0.8 mmol/L CuSO_4_ treatment groups, respectively (fig 2B). *ctl-2* encodes a peroxisomal catalase, the mRNA level of which was increased by approximately 3.2-fold and 2.2-fold in the 0.4 and 0.8 mmol/L CuSO_4_ treatment groups, respectively (fig 2C). *ctl-3* encodes a tissue specific catalase; as expected, the mRNA levels of *ctl-3* were not significantly up-regulated in any of the CuSO_4_ treatment groups. These results indicate that *ctl-1* and *ctl-2* are induced by copper stress. A similar induction was also observed during lead exposure in nematodes [49].

### GPX activity and mRNA expression levels

**Figure 3:**
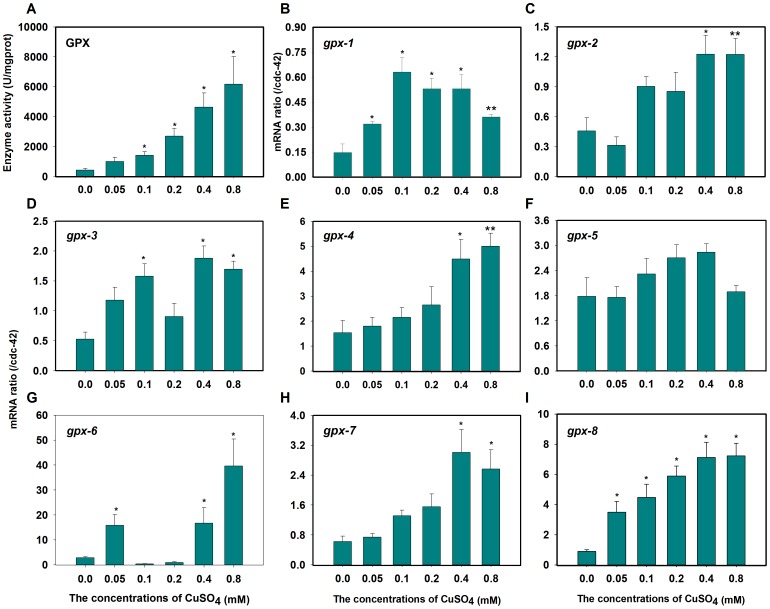
GPX activities or mRNA expression induced by copper in *C. elegans*. (A) The GPX activity was elevated when exposed to copper. Values are presented as the means ±SE (*n* = 3) in U mg^−1^ Pr. (B) gpx-1, (C) gpx-2, (D) gpx-3, (E) gpx-4, (F) gpx-5, (G) gpx-6, (F) gpx-7, and (G) gpx-8 expression ratios were each quantified in nematode N2 at different concentrations of copper and normalized with *cdc-42*. *P<0.05, **P<0.01 versus respective controls analyzed by *t*-test.

Limited evidence for the role of GPX in organismal copper detoxification has been documented [5], [50]. The results in fig 3A show that GPX activity increased in a dose-dependent manner in *C. elegans*. Significant increases were observed in all CuSO_4_ treatments, after which GPX levels were approximately 1.3-, 2.3-, 5.2-, 9.7-, and 13.3-fold greater than those of controls. Additionally, increased GPX activity plays a central role in protecting against the deleterious effects of copper in the blue mussel [50]. Our results indicate that concentrations as low as 0.05 mmol/L CuSO_4_ might initiate physiological damage.

In worms, eight homologs of human GPX genes have been identified. qPCR results showed that mRNA levels of almost all GPX genes, excluding *gpx-5*, could be increased by copper exposure in *C. elegans* (fig 3). The induction of *gpx-1* was increased by 2.2-, 2.3-, 3.6-, 3.6-, and 2.4-fold, respectively, in the 0.05, 0.1, 0.2, 0.4, 0.8 mmol/L CuSO_4_ treatment groups. The patterns of *gpx-2*, *gpx-3*, *gpx-4*, *gpx-6*, and *gpx-7* expressions were similar, with maximum induction appearing in the 0.4 and 0.8 mmol/L treatment groups (fig 3 C, D, E, G, and H). The induction of *gpx-8* was dose-dependent and significantly increased in all treatment groups (fig 3I). *gpx-5* expression levels did not change compared to those in controls (fig 3F). These results suggest that GPX genes may play an important role in the regulation of copper detoxification. These elevations were also observed in HepG2 cells under conditions of copper stress [5].

Although the activation of the antioxidant system consisting of SOD, CAT, and GPX can be rather complex, the desired effects of neutralizing free radicals and their toxic effects occur in a multi-step process [10]. However, a dose-dependent decrease in GST was also observed in the worms. The maximum decreases occurred at the treatment levels of 0.4 and 0.8 mM (88% and 79%, respectively) (fig S1). The decrease in GST may be due to the toxic effects of copper, despite the ready availability of other cellular defenses. Additionally, such a decrease may have occurred before the copper-dependent induction of degenerative processes [47]. These results indicate that copper stress induces SOD, CAT, and GPX activities in *C. elegans*. Moreover, *myo*-inositol (MI), a nutrient antioxidant, mediates increases in SOD, CAT, and GPX (not including GST) that contribute to lipid and protein copper oxidant repair [51]. For this reason, our attention was mainly directed toward these three major enzymes, which act jointly in the destruction of ROS in the worm.

### LC_50_ values of genetic strains in response to CuSO_4_

To determine whether the genes induced by copper are necessary for copper detoxification processes, we detected the LC_50_ of CuSO_4_ in genetic loss-of-function mutants and some strains with representative over-expressed mRNA at L_4_ stages. Because the bags-of-worms phenotype would be induced by the exposure of 3-days-old worms (young adult) to copper for a 24 h period, and Live/dead scoring would be impaired by the fact that the eggs would hatched inside the mother. Our results showed that the loss-of-function mutants *sod-5, ctl-1*, *gpx-3*, *gpx-4*, and *gpx*-6 were significantly more sensitive to CuSO_4_ compared to controls (table 2). Among the six mutants, *ctl-1* and *gpx-3* were the most sensitive. Interestingly, the transcription of all of six genes was induced during copper stress conditions. A lack of hypersensitivity in other copper -responsive gene mutant strains (such as: *sod-3 (tm760)*, and *ctl-2 (ok1137)*) suggests that compensatory or other factors may be involved in the response to copper. The lack of severe oxidative stress hypersensitivity when *sod-2*, and *sod-3* are deleted was attributed to the compensatory effects of increase in function. Protein and lipid damage has also not detected in the *sod-1* mutant [46]. The identity of compensatory factors remains to be determined. In addition, the over-expression of *sod-1*, *sod-2*, *sod-3*, and *ctl-1+ctl-2+ctl-3* resulted in 1.4- to 2.5-fold increases in the LC_50_ of CuSO_4_ over that of the wild type (table 2). The overexpression of *sod-1* increased the worm's life span, and total SOD activity was increased by approximately two- fold by *wuIs152* [46]. Thus, the over-expression of antioxidant enzyme may suppress copper toxicity.

**Table 2:** The LC_50_ values of each strain for CuSO_4_ (concentration in mmol/L).

Strains	LC_50_ (95% CI^a^)	Strains	LC_50_ (95% CI)
N2	0.88 (0.84–0.92)	*gpx-1* (tm2100)	0.81 (0.72–0.90)
*sod-1* (tm776)	0.81 (0.74–0.89)	*gpx-2* (tm2895)	1.03 (0.81–1.27)
*sod-2* (gk257)	1.05 (0.90–1.20)	*gpx-3* (tm2139)	0.52 (0.48–0.55)
*sod-3* (tm760)	0.88 (0.77–1.00)	*gpx-4* (tm2111)	0.66 (0.62–0.70)
*sod-4 (gk101)*	0.84 (0.75–0.93)	*gpx-5* (tm2024)	0.79 (0.72–0.87)
sod-5 (tm1146)	0.76 (0.70–0.83)	*gpx-6* (tm2535)	0.67 (0.61–0.73)
*ctl-1* (ok1242)	0.44 (0.34–0.53)	*gpx-7* (tm2166)	0.87 (0.79–0.96)
*ctl-2* (ok1137)	0.91 (0.73–1.05)	*gpx-8* (tm2108)	0.91 (0.83–0.98)
*ctl-3* (ok2042)	0.99 (0.92–1.05)	wuIs152 (sod-1gDNA)	1.58 (1.44–1.76)
*wuIs156* (*sod-2*gDNA)	2.12 (1.95–2.31)	*huIs33* (*sod-3*gDNA)	1.21 (1.15–1.27)
*wuIs151*(*ctl-1*+*ctl-2*+*ctl-3*+myo-2::GFP)	1.32 (1.15–1.49)	*daf-16 (m26)*	0.85 (0.78–0.91)
*age-1* (hx546)	1.62 (1.52–1.72)	*daf-2* (e1368)	2.23 (1.84–2.77)
*daf-16*(m26);*age-1*(m333)	0.82 (0.68–0.97)	*daf-16*(m26);*daf-2*(e1370)	1.62 (1.38–1.86)

Note: ^a^, 95% confidence interval.

The LC_50_ value of N2 in this study was not exactly the same as that reported in Tatara's study [42]; this discrepancy may have occurred because the L_4_ larvae stage was used in the present study, whereas young adults were used in the previous study [42]. The *wuIs152* –driven (overexpression of SOD-1) further decreased sensitivity in copper did not occur in the same manner as similar increases in paraquat sensitivity [46]. *sod-1* deletion mutant also increased sensitivity to paraquat [46], but not to copper. This result may be due to the slower transition of copper-generated O^2−^ into H_2_O_2_ in copper than paraquat, as toxic processes are different between the two compounds. The observation that CAT over-expression causes copper hyposensitivity is supported by experiments of CAT over-expression in Drosophila [52]. The strain *gpx-1 (tm2100)* showed no significant change in the LC_50_ to CuSO_4_, which is supported by the fact that the mitochondrial ROS load was not affected in these strains [53]. It was concluded that *sod-5*, *ctl-1*, *gpx-3*, *gpx-4*, and *gpx-6* might be necessary for the copper detoxification process, although other genes for encoding antioxidant enzymes might also participate in the process.

### The *daf-2* and *age-1* mutants reduced copper sensitivity

A recent study implicated insulin/IGF-1 mutants are associated with stress response in nematodes as negative regulators of antioxidant enzymes [28], [30]. Consistent with the model that mutations in *daf-2* and *age-1* cause reduced sensitivity to heat, oxidants, UV, and heavy metals [28], [30], both *daf-2* and *age-1* mutants reduced sensitive to copper stress, as reflected by their increased survival LC_50_ values (table 2). However, the increased resistance of *age-1* and *daf-2* to copper is reduced by constructing double mutants with *daf-16*. Both *daf-16;daf-2* and *daf-16;age-1* exhibited increased copper sensitivity compared with *daf-2* and *age-1* single mutants (table 2). These results indicate that the reduced copper sensitivity in *daf-2* and *age-1* mutants is mediated by *daf-16*. Interestingly, the LC_50_ value more substantially reduced the sensitivity of *daf-2* mutant than that of *age-1* (table 2). These effects may be caused by reduced insulin/IGF-1 signaling or they may be mediated by a distinct pathway emanating from DAF-2 that does not involve PI-3-kinase [54]. Consistent with previous findings, the sensitivity of *daf-16* mutant to copper is similar to that of wild-type nematodes [28]. Double mutants with *unc-75* have a 3-fold increased sensitivity to copper [55]. These results suggested the presence of another stress protection pathway, possibly *unc-75* [55].

### SOD, CAT, and GPX activities in *daf-2* and *age-1* mutants

**Figure 4:**
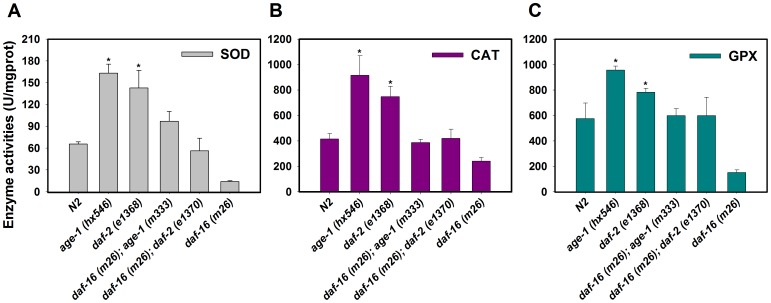
Antioxidant enzymes in *daf-2*, and *age-1* mutants. (A) SOD activity in wild-type and *daf-2*, *age-1*, *daf-16;daf-2*, *daf-16;age-1*, *daf-16* mutants. (B) CAT activity in these six strains. (C) GPX activity in these six strains. Values are presented as the means ±SE (*n* = 3) in U g^−1^ Pr. *P<0.05, **P<0.01 versus respective controls analyzed by *t*-test.

Elevated activities of antioxidant enzymes in worms were present in the *daf-2* and *age-1* mutants relative to wild-type worms (fig 4). SOD and CAT activities were approximately twice as great as those measured in wild-type worms (fig 4A and B). GPX activity was also elevated in both mutant strains (*daf-2* and *age-1*) (fig 4C), and this elevation was counteracted in double mutants with *daf-16(m26)*. The activation of SOD, CAT, and GPX was decreased in the *daf-16* single mutant. The elevated SOD and CAT levels observed here are consistent with previous studies [30], [56], and this is the first observation of elevated GPX in these mutants. The up-regulation of SOD, CAT, and GPX may be frequently associated with increased copper resistance (table 2 and fig 4). This hypothesis is also supported by the role of *daf-2* and a*ge-1* gene products as negative regulators of SOD and CAT activities [57].

### mRNA expression levels of *sod*, *ctl*, and *gpx* in *daf-2* and *age-1* mutants

**Figure 5:**
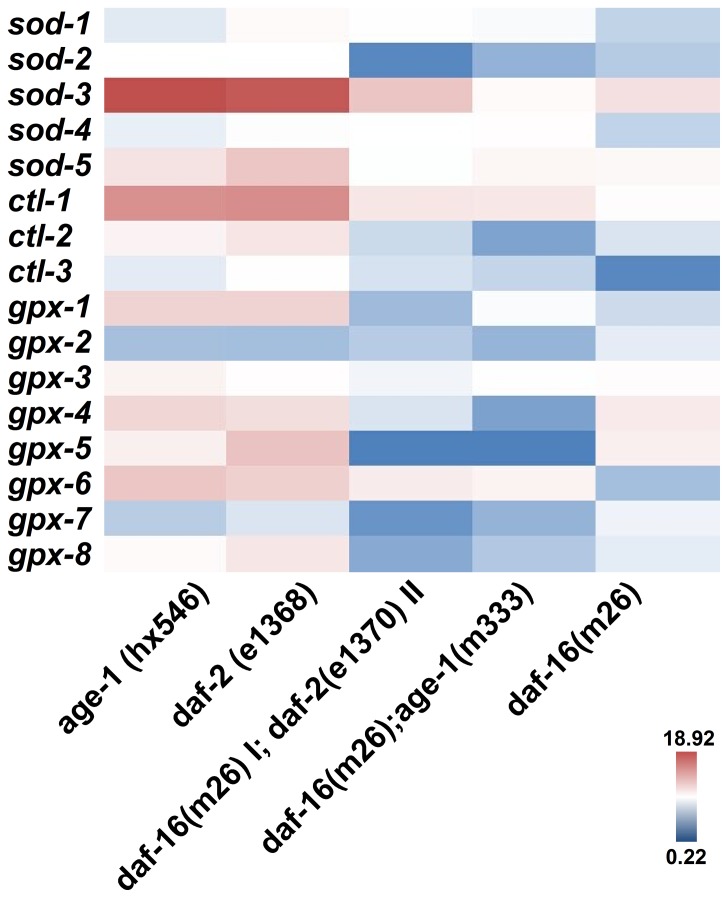
mRNA expression levels of antioxidant enzymes in *daf-2*, *age-1*, *daf-2;daf-16*, *age-1;daf-16* and *daf-16* mutants. Heat map analysis of the RNA expression patterns of *daf-2* and *age-1* versus N2. Both *sod-3* and *sod-5* expression levels were increased in *daf-2* and *age-1* mutants. Both *ctl-1* and *ctl-2* were increased in *daf-2* and *age-1* mutants. *gpx-3,4,5,6,7,8* were increased in *daf-2* and *age-1* mutants. There were no significant difference between N2 and *daf-16* mutant except for *sod-3* and *gpx-4*. The red is high; the blue is low.

Fig 5 shows the mRNA expression of *sod*, *ctl*, and *gpx* in *daf-2, age-1*, *daf-2;daf-16*, and *age-1;daf-16* mutants compared with controls during K-medium cultivation. In *daf-2* and *age-1* mutants, there was a significant increase in *sod-3* (nearly 18- and 17-fold, respectively), *sod-5* (approximately 4-and 7-fold, respectively), *ctl-1* (approximately 12- and 12-fold, respectively), *ctl-2* (approximately 2- and 3-fold, respectively), *gpx-1* (approximately 2- and 3-fold, respectively), *gpx-3* (approximately 3- and 2-fold, respectively), *gpx-4* (approximately 5- and 4-fold, respectively), *gpx-5* (approximately 3- and 7-fold, respectively), *gpx-6* (approximately 7- and 6-fold, respectively), and *gpx-8* (nearly 2- and 4-fold, respectively) expression. These elevations were partially attenuated when these mutations were constructed as double mutants with *daf-16*. The expressions of *sod-1*, *sod-2*, *sod-4*, *ctl-3*, *gpx-2*, and *gpx-7* did not change in these mutants. Only the expressions of *sod-3* and *gpx-4* in all tested genes were significantly different in *daf-16* mutant compared with wild type. The results of the *sod* and *ctl* were consistent with previous reports [29], [58].

### Responsive capacities of antioxidant enzymes to copper in the *daf-2* mutant

**Figure 6:**
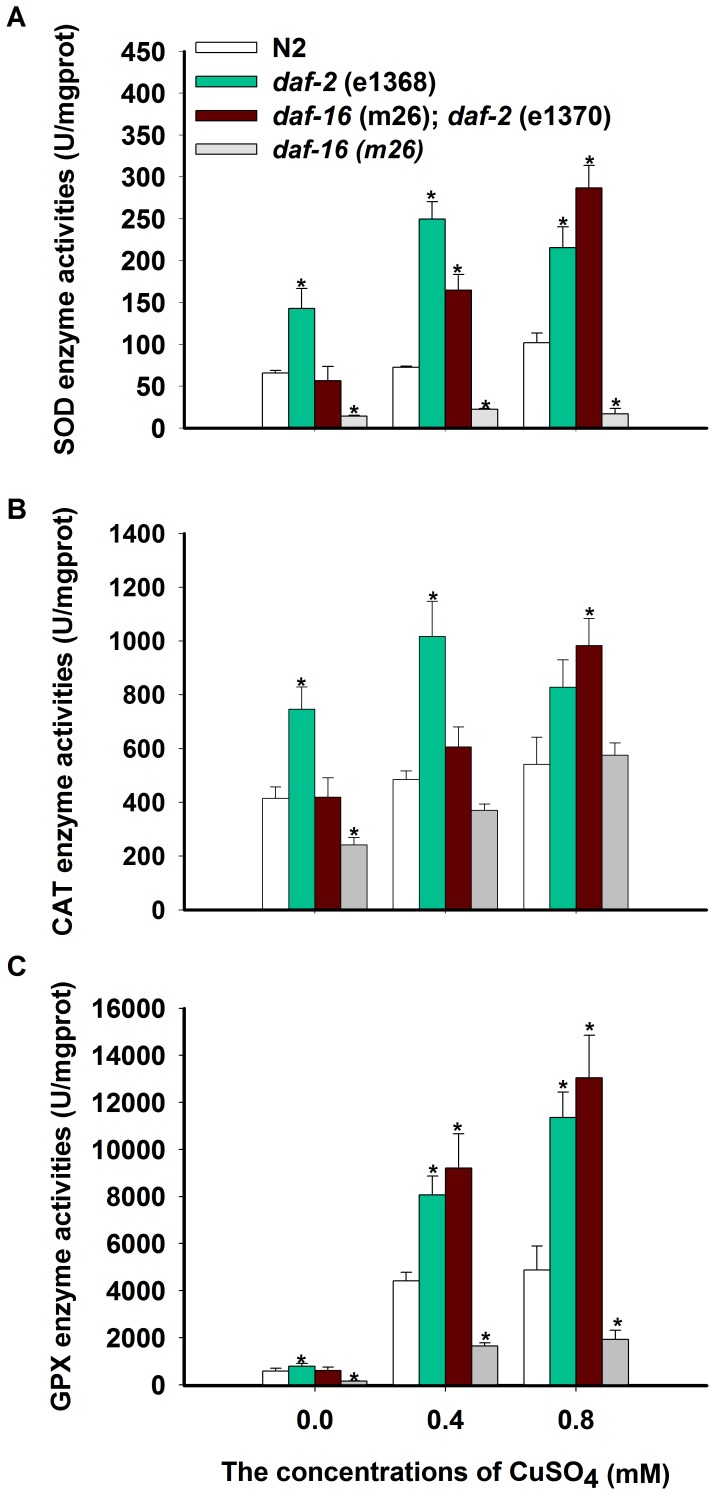
Antioxidant enzymes were induced by copper in *daf-2*, *daf-16*, and *daf-16;daf-2* mutants. The activities of SOD (A), CAT (B), and GPX (C) were quantified in nematode N2, *daf-2*, *daf-16*, and *daf-16;daf-2* treated with different concentrations of copper. All values are given as the means ±SE (*n* = 3) in U g^−1^ Pr. *P <0.05, **P<0.01 versus respective controls analyzed by *t*-test.

SOD, CAT, and GPX activities increased steadily with copper-treated wild-type animals. Such increases were also observed in the insulin/IGF receptor mutant *daf-2* and transcription factor *daf-16* (fig 6). Whereas SOD activity increased by 1.1- and 1.6-fold at 0.4 and 0.8 mM CuSO_4_, respectively, in wild-type worms, it increased by 1.7- and 1.5-fold in the *daf-2* mutant, 2.9- and 5.1-fold in the *daf-16;daf-2* mutant and 1.1- and 1.6- fold in *daf-16* mutant. Whereas CAT activity increased by 1.2-fold and 1.3- fold at 0.4 and 0.8 mM CuSO_4_, respectively, in wild-type worms, it increased by 1.4 and 1.1- fold in *daf-2* mutants, 1.4- and 2.3- fold in the *daf-16;daf-2* mutant and 1.5- and 2.5- fold in *daf-16* mutants. Whereas the GPX activities increased by 7.7-fold and 8.4-fold at 0.4 and 0.8 mM CuSO_4_, respectively, in wild-type worms, it increased by 10.3-fold and 14.5-fold in the *daf-2* mutant, 15.4-fold and 21.8-fold in the *daf-16;daf-2* mutant, and 6.7- and 20.6-fold in the *daf-16* mutant compared to wild-type worms.

Moreover, these results demonstrated the greater potential for simultaneous up-regulation of SOD, CAT and GPX activities in *daf-2*, *daf-16;daf-2*, and *daf-16* mutants than in wild-type worms exposed to CuSO_4_ (fig 6). The underlying mechanism was likely that reduced insulin/IGF-1 signaling relieved inhibitory actions against the antioxidant enzymes, eliciting the coordinated expression of an elaborate detoxification program. The present results not only support the free radical theory, which states that copper induces oxidative enzymes to protect organisms against ROS damage through a Fenton-like reaction [12], but also indicate that increased oxidative enzyme activities may be among the causes of increased hyposensitivity to copper in both *daf-2* and *age-1* mutants. *daf-2* displayed the greatest increases in GPX activity when exposed to copper compared to the three wild-type enzyme activities. This result indicates that additional signaling pathways may contribute to these processes. It was reported that AP-1, JNK/SAPK, NF-kappaB signaling, nrf and hepatocyte nuclear factor 4-alpha might participate in controlling copper-responsive transcription in human cells [3], [7], [8], [59].

### Induction of *sod*, *ctl*, and *gpx* mRNAs expression by copper in *daf-2, age-1*, and *daf-16* mutants

**Figure 7:**
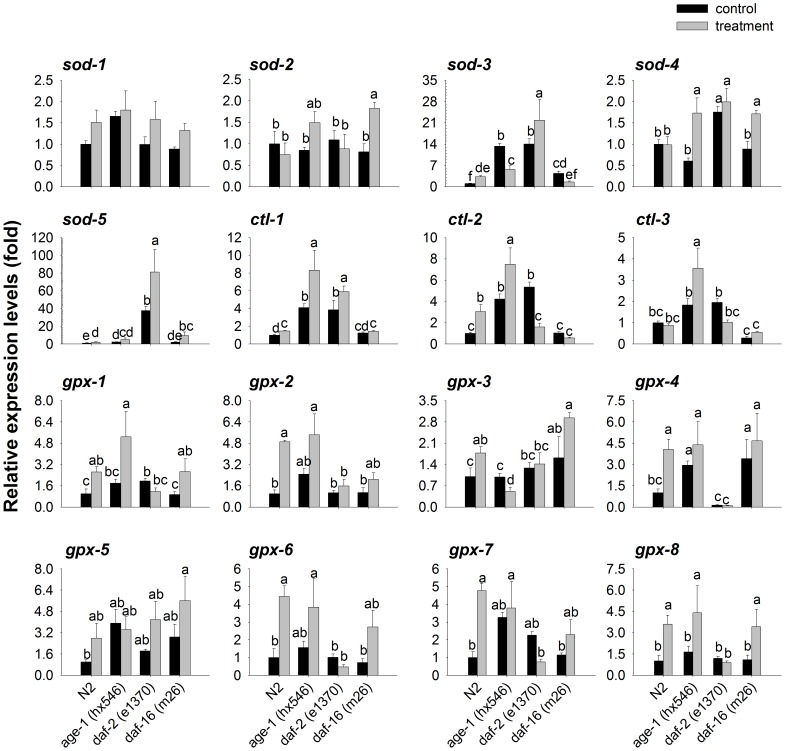
mRNA expression levels of antioxidant enzymes response capacities to copper in *daf-2*, *age-1*, *daf-16* mutants. The mRNA expression levels of *sod*, *ctl*, and *gpx* were quantified in nematode N2, *daf-2*, *age-1*, and *daf-16* treated with 0.8 mM CuSO_4_. All values are given as the means ±SE (*n* = 4). Lower case represents significantly difference, *P*<0.05, analyzed by Duncan's multiple comparison test.

Initially the strains were exposed to a toxic concentration of LC_50_ CuSO_4_ (nominal value). After treatment with CuSO_4_, the mRNA expression levels of *ctl-1*, and *sod-5* increased in all strains tested. The highest increases of mRNA expression levels (>4-fold; *P*<0.01) were occurred in *gpx-2*, *gpx-4*, *gpx-6*, and *gpx-7* in wild-type worms and in *sod-5* in *daf-16* mutants. Smaller but still significant increases (>3-fold; *P*<0.01) were observed in *sod-3*, *ctl-2*, and *gpx-8* in wild-type worms and *gpx-8* in *daf-16* mutants (fig 7). We only extracted mRNA from live animals. At 0.8 mM Cu 50% of the wild-type and *daf-16* nematodes were dead, but only ∼20–30% of *daf-2* and *age-1* mutants were dead. Consequently, selection for the more resistant wild-type and *daf-16* worms might be taking place in these experiments. And This process might be one reason why the highest increases in mRNA expression levels were not found in *daf-2* and *age-1* mutants.

The tested gene expression level profiles of *daf-16* mutants were similar to those of wild-type worms under basal conditions. And the increased mRNA expression level of *sod-3*, *ctl-1*, *ctl-2*, *gpx-1*, *gpx-2*, *gpx-3*, *gpx-4*, *gpx-6*, *gpx-7*, which were copper -response genes, were not occured in *daf-16* mutant when treated with copper. It indicate that these genes might be the target of daf-16-mediated transcription. The mRNA levels of *sod-3* and *sod-5* in copper -exposed *age-1* mutants were intermediate between those of wild-type worms and *daf-2* mutants (fig 7). This factor might be the underlying reason why *daf-2* mutant are more resistant than *age-1* to copper. But it is still uncertain to what extent oxidative stress contributes to their resistant to copper.

### Conclusions

Overall, copper induces a complex regulation of antioxidant enzymes. In the present study, we identified copper-responsive antioxidant enzymes and genes. Of these, *sod-5*, *ctl-1*, *gpx-3, gpx-4, gpx-6* were necessary for copper detoxification. Other genes also played a role in copper detoxification. *daf-2* and *age-1* mutants were more resistant to copper compared to wild-type worms and also revealed the up-regulatory capacity of antioxidant enzyme activity and expression during copper treatment. The present study provides evidence of the beneficial effects of antioxidant enzymes during copper detoxification in model organisms as well as a better understanding of the utility of *C. elegans* as a model for the study of metal detoxification mechanisms in humans.

## Supporting Information

Figure S1 **GST activities were decreased by copper in** ***C. elegans*** **.** GST activity in nematodes N2 at different concentrations of copper exposure. All values are given as the means ±SE (*n* = 3) in U mg^−1^ Pr.
(TIF)

Table S1 Primer Sequences for qPCR.
(DOCX)
