# Supplementary figures and images for: Molecular Basis for Antioxidant Enzymes in Mediating Copper Detoxification in the Nematode Caenorhabditis elegans
Source: PLoS One. 2014 Sep 22;9(9):e107685. doi: 10.1371/journal.pone.0107685 (PMC4171499; doi:10.1371/journal.pone.0107685)

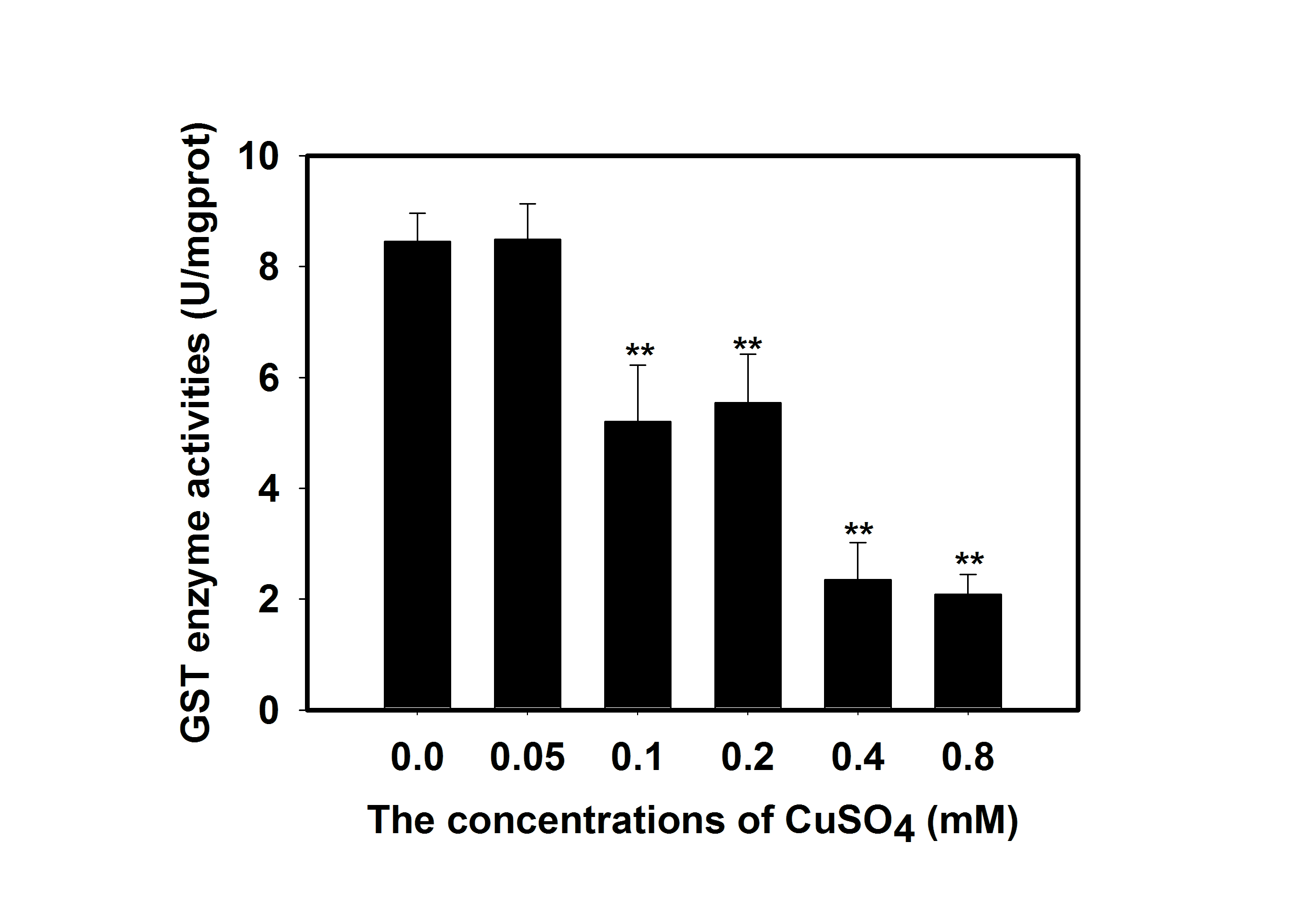

Supplement: Figure S1 — GST activities were decreased by copper in C. elegans . GST activity in nematodes N2 at different concentrations of copper exposure. All values are given as the means ±SE (n = 3) in U mg−1 Pr. (TIF) [file pone.0107685.s001.tif]
