# Supplementary material for: Molecular Basis for Antioxidant Enzymes in Mediating Copper Detoxification in the Nematode Caenorhabditis elegans
Source: PLoS One. 2014 Sep 22;9(9):e107685. doi: 10.1371/journal.pone.0107685 (PMC4171499; doi:10.1371/journal.pone.0107685)
Supplement: Table S1 — Primer Sequences for qPCR. (DOCX) [file pone.0107685.s002.docx]

Table S Primer sequences for qPCR

| **Gene** | **Forward primer** | **Reverse primer** | **Amplicon size** |
| --- | --- | --- | --- |
| ***cdc-42*** | CTGCTGGACAGGAAGATTACG | CTCGGACATTCTCGAATGAAG | 115 |
| **Y45F10D.4** | GTCGCTTCAAATCAGTTCAGC | GTTCTTGTCAAGTGATCCGACA | 139 |
| ***sod-1*** | CGTAGGCGATCTAGGAAATGTG | TGACGAGCGTGTCGGTGAG | 74 |
| ***sod-2*** | GATACTGTCCAAAGGGAAAGAT | GTAGTAAGCGTGCTCCCAGA | 119 |
| ***sod*-*3*** | ATCTACTGCTCGCACTGCTT | TTTCATGGCTGATTACAGGTT | 128 |
| ***sod*-*4*** | GCACCAGATGACTCGAACA | GTCCACTTAATGAGGCAAGA | 109 |
| ***sod-5*** | TCGAAACGTGCTGTAGCGG | CACCTTCGGCTTTCTGGGT | 76 |
| ***ctl-1*** | AGTGATTTGGGTGGCTGTT | AATGTACAAATTCCGTTTAATG | 133 |
| ***ctl-2*** | CTGGGAGAAGGTGTTGGAT | GGATGAACCTTTGAAAAGTGAT | 123 |
| ***ctl-3*** | AGTAAATCTTCAAAATGCCAATG | GGTGGGGTTCCTGATTTCTAT | 123 |
| ***gpx-1*** | AACGACTGATCCAAAGGAC | TTGAAAGTTTGAATTGCTG | 87 |
| ***gpx-2*** | GACTACTGAGCCGAAGGAT | CCAAGTTTTCACTCGATTTT | 110 |
| ***gpx-3*** | ATCTGGTAACATGGCACCT | TCCAACGCATTGTTTCATC | 104 |
| ***gpx-4*** | TACTTACTTTGGCTGTTTCTTTCAC | TTTCCAGCGCAGAGTATCG | 77 |
| ***gpx-5*** | TCGTAGTACCGACCCACAT | GTAAATACAGGAACGGAGAAAA | 121 |
| ***gpx-6*** | GCCAGATATGTATTCAAAGG | GCTGATAATGATGAGCCAC | 173 |
| ***gpx-7*** | GGCCATTGATTGGAGAAGA | CCATTTGATTGCATCGAAAA | 89 |
| ***gpx-8*** | TGGCGAATGAAACAGTAAA | GACAGTCGTTGATCTATGC | 95 |
